# LocPro: A deep learning-based prediction of protein subcellular localization for promoting multi-directional pharmaceutical research

**DOI:** 10.1016/j.jpha.2025.101255

**Published:** 2025-03-05

**Authors:** Yintao Zhang, Lingyan Zheng, Nanxin You, Wei Hu, Wanghao Jiang, Mingkun Lu, Hangwei Xu, Haibin Dai, Tingting Fu, Ying Zhou

**Affiliations:** a Department of Pharmacy, Second Affiliated Hospital, Zhejiang University School of Medicine, Hangzhou, 310009, China; b College of Pharmaceutical Sciences, Zhejiang University, Hangzhou, 310058, China

**Keywords:** Protein subcellular location, Pharmaceutical research, Protein large language model, Multi-label prediction

## Abstract

Drug development encompasses multiple processes, wherein protein subcellular localization is essential. It promotes target identification, treatment development, and the design of drug delivery systems. In this research, a deep learning framework called LocPro is presented for predicting protein subcellular localization. Specifically, LocPro is unique in (a) combining protein representations from the pre-trained large language model (LLM) ESM2 and the expert-driven tool PROFEAT, (b) implementing a hybrid deep neural network architecture that integrates convolutional neural network (CNN), fully connected (FC) layer, and bidirectional long short-term memory (BiLSTM) blocks, and (c) developing a multi-label framework for predicting protein subcellular localization at multiple granularity levels. Additionally, a dataset was curated and divided using a homology-based strategy for training and validation. Comparative analyses show that LocPro outperforms existing methods in sequence-based multi-label protein subcellular localization prediction. The practical utility of this framework is further demonstrated through case studies on drug target subcellular localization. All in all, LocPro serves as a valuable complement to existing protein localization prediction tools. The web server is freely accessible at https://idrblab.org/LocPro/.

## Highlights

• A dual-channel protein representation approach combining ESM2 and expert-driven features.
• A hybrid deep neural network architecture combining CNN, FC, and BiLSTM blocks.
• A multi-label framework for predicting protein subcellular localization at multiple granularity levels.

## Graphical abstract

**Figure:**
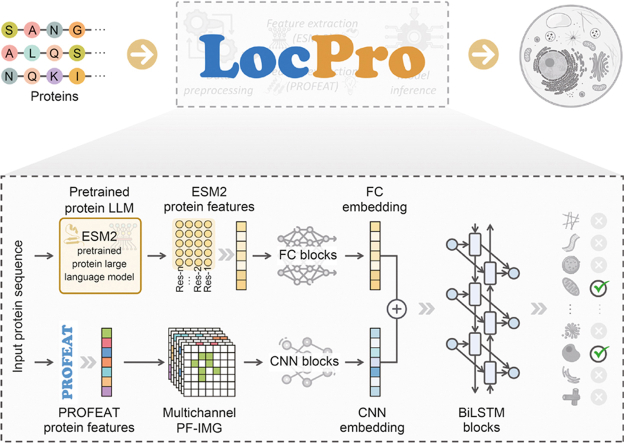

## 1 Introduction

The subcellular localization of proteins plays a critical role in drug discovery and development. Aberrant protein localization has been implicated in various pathological conditions [1], including cancers [2], cardiovascular diseases [3], and neurodegenerative disorders [4]. Understanding protein subcellular localization is essential for the identification of therapeutic targets [5], the development of novel treatments [6], and the design of drug delivery systems [7], among other advancements. While experimental approaches such as microscopy-based methods [8] and proximity labeling techniques [9] provide reliable determination of protein subcellular localization, these methods typically require specialized equipment and are time-consuming and labor-intensive. Given these constraints and the rapidly expanding volume of protein data [10,11], computational methods for predicting protein subcellular localization have emerged as valuable complementary tools.

Several approaches have been developed for predicting protein subcellular localization. Some methods focus on predicting localization within specific organelles [12], which limits their applicability to individual organelles rather than providing a comprehensive whole-cell prediction. Other approaches are species-specific [13], restricting their generalizability across different organisms. Additionally, some studies treat protein localization prediction as a multi-class classification task for single localization [14], overlooking the phenomenon of multi-localization. Currently, widely used protein sequence-based tools, such as DeepLoc 2.0 [15] and MULocDeep [16], provide multi-label predictions for protein subcellular localization at the whole-cell level. However, DeepLoc 2.0 predicts 10 major subcellular localizations, while MULocDeep includes these 10 categories along with 44 additional sub-organellar localizations. Both tools impose restrictions on protein sequence length, analyzing only the terminal regions of longer protein. Therefore, developing a comprehensive tool for protein subcellular localization prediction that addresses these limitations represents a significant and valuable research challenge.

In recent years, the advancement of deep learning has led to the widespread application of representation learning in biomedical research [17,18]. The goal of representation learning is to automatically extract meaningful feature representations from unlabeled data, thereby facilitating downstream tasks. For example, protein language models such as ESM2 [19], ProteinBERT [20], and ProtT5 [21], through unsupervised pretraining on large-scale protein sequence datasets, have demonstrated the ability to capture latent structural, functional, and evolutionary information embedded in amino acid sequences [22]. Furthermore, several studies have demonstrated that combining expert-driven features with pre-trained models can enhance performance [23]. PROFEAT is a comprehensive expert-driven protein sequence representation tool that provides 1,484 descriptors across seven categories [24] (as shown in Table S1). Therefore, both ESM2 and PROFEAT are utilized for the research.

**Fig. 1:**
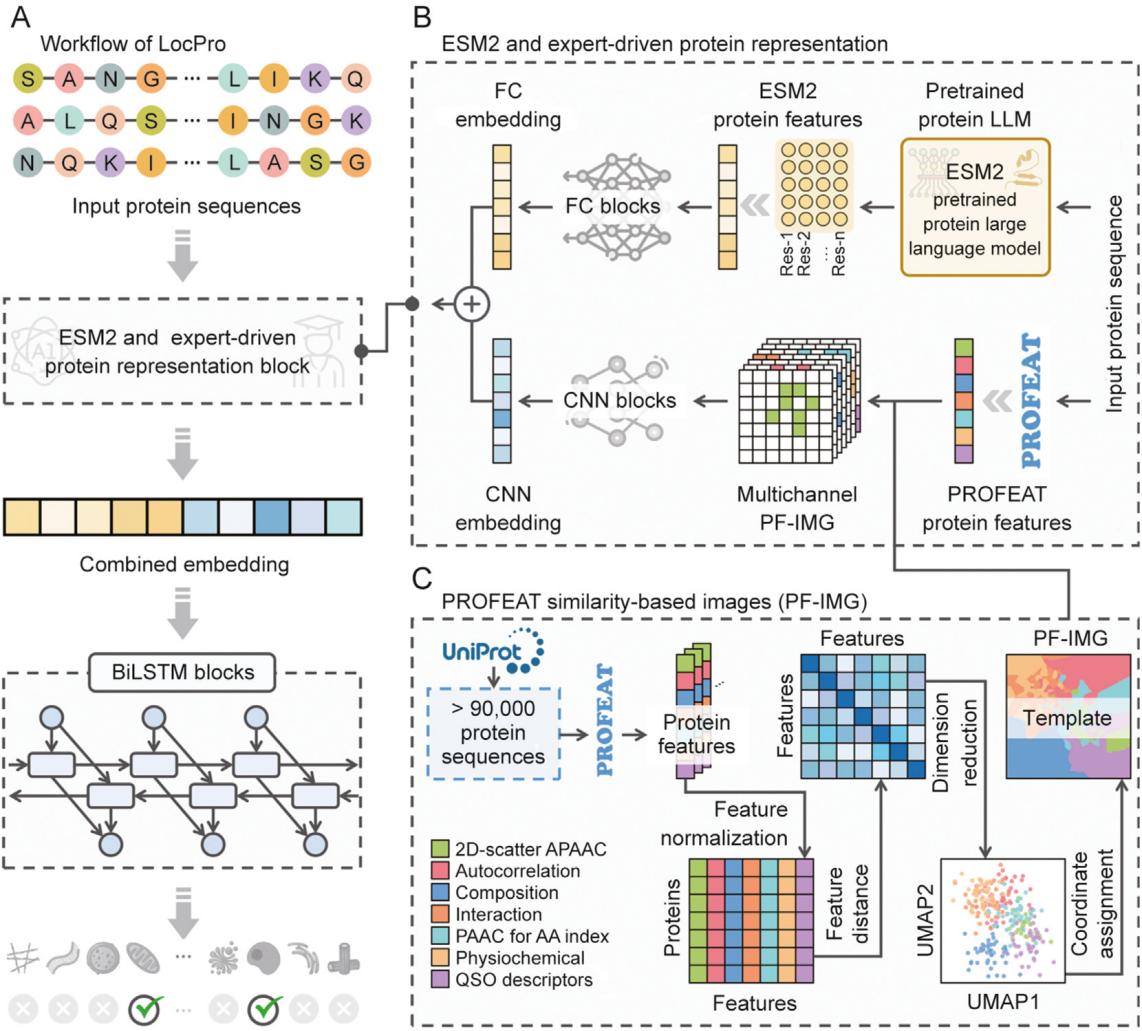
Overview of the LocPro framework for protein subcellular localization prediction. (A) Workflow of LocPro. Input protein sequences are processed through the ESM2 and expert-driven protein representation block, which generates and integrates embeddings from two different sources. These embeddings are then passed through bidirectional long short-term memory (BiLSTM) blocks, ultimately feeding into a multi-label classifier that predicts specific subcellular localizations. (B) ESM2 and expert-driven protein representation. This section consists of two parallel pathways: (1) Global features based on ESM2 are extracted from the input sequence and subsequently transformed through fully connected (FC) blocks to generate FC embedding. (2) PROFEAT-based features are generated and rearranged, followed by transformation through convolutional neural network (CNN) blocks to produce CNN embedding. (C) PROFEAT similarity-based images (PF-IMG). The construction of PF-IMG involves calculating PROFEAT features for over 90,000 protein sequences, assessing feature similarities, performing dimensionality reduction, and rearranging the reduced feature space into a two-dimentional (2D) grid image template. APAAC: amphiphilic pseudo amino acid composition; PAAC: pseudo amino acid composition; QSO: quasi-sequence-order; UMAP: uniform manifold approximation and projection.

A hybrid deep learning framework, named LocPro, was developed for protein subcellular localization prediction. As illustrated in Fig. 1, our approach incorporates several key components. First, ESM2 was utilized to obtain global protein sequence representations, which effectively capture amino acid sequence characteristics while reducing dimensionality [25]. Second, unsupervised similarity computation and reorganization of PROFEAT-engineered features to further extract correlational information between representations [26]. Third, a hybrid deep neural network by integrating convolutional neural network (CNN), fully connected (FC) layer, and bidirectional long short-term memory (BiLSTM) blocks to process these features. The effectiveness of our novel approach was validated through comparisons with popular existing tools. Overall, LocPro demonstrated excellent performance, establishing itself as a valuable complement to existing protein subcellular localization prediction methods.

## 2 Materials and methods

### 2.1 Model design and implementation

#### 2.1.1 Workflow of LocPro

The LocPro framework, as depicted in Fig. 1A, follows a five-step sequential workflow. Designed as an end-to-end model, LocPro begins by accepting protein sequences as input. The second step incorporates a dual-channel representation block that combines ESM2 and expert-driven features. This block simultaneously extracts global sequence representations using the pre-trained protein language model ESM2 and generates feature representations through PROFEAT-based engineering. In the third step, these dual-channel protein representations are integrated into a unified feature set. The fourth step involves processing this integrated representation through BiLSTM blocks, and the final step applies a multi-label prediction classifier to generate subcellular localization predictions. The framework addresses protein localization at two levels: 10 major subcellular localizations and 91 subcellular localizations at varying levels of granularity. The source code for the LocPro tool is publicly available on GitHub (https://github.com/idrblab/LocPro) under the MIT license. Additionally, a web-based interface for LocPro predictions has been implemented and is now freely accessible to all users at https://idrblab.org/LocPro/.

#### 2.1.2 ESM2 and expert-driven protein representation

The ESM2 and expert-driven protein representation block, as depicted in Fig. 1B, consists of two distinct methodological approaches. The ESM2 channel processes protein sequences to generate 1,280-dimensional per-residue vectors, which are then aggregated via average pooling to construct global representations. These global representations are subsequently transformed through FC blocks into embeddings. In this study, the 650 million parameter version of ESM2 was utilized to generate embeddings for protein sequences. For particularly long protein sequences, a segmentation approach was employed, where the sequences were divided into segments, and embeddings were generated for each segment. The final embedding for the entire sequence was obtained by taking a weighted average of the segment embeddings. The relevant formulas and additional details are provided in Section Method S1 of Supplementary data and Table S2. Meanwhile, the PROFEAT channel generates expert-driven 1,484-dimensional feature vectors, which are restructured according to the PROFEAT similarity-based images (PF-IMG) template. These vectors are then processed through CNN blocks to obtain corresponding embeddings.

#### 2.1.3 PF-IMG

The process of generating the PF-IMG template, as illustrated in Fig. 1C, involves several sequential steps. First, based on the CAFA dataset [27], over 90,000 protein sequences from UniProt [28] were obtained. These sequences were processed using PROFEAT to generate 1,484-dimensional feature vectors for each protein, covering seven distinct categories (as detailed in Table S1). The resulting protein features were then consolidated into a protein-feature matrix. This matrix underwent a “Feature normalization” step, utilizing techniques such as Min-Max Normalization [29]. The next step involved calculating “Feature distance” using methods like Cosine Distance [30] to create a feature distance matrix. Following this, “Dimension reduction” was performed using algorithms such as uniform manifold approximation and projection (UMAP) [31] to generate a low-dimensional clustering visualization of the features. Finally, a “Coordinate assignment” step was conducted, employing methods such as the J-V algorithm [32], to redistribute the features onto a two-dimentional (2D) grid, ultimately resulting in the PF-IMG template. The relevant formulas and additional details are provided in Section Method S2 of Supplymentary data.

### 2.2 Model parameters and optimization strategy

LocPro integrates deep learning architectures in a sophisticated manner, combining CNN, FC, and BiLSTM networks within an optimized multi-module structure. The framework processes protein features through two parallel pathways. One pathway utilizes native ESM2-based embeddings, which are processed through two FC layers. The other pathway employs PROFEAT-engineered features, which are analyzed using CNN blocks consisting of dual convolutional layers with 3 × 3 kernels (stride of 2) and two max-pooling layers (pool size of 2, stride of 2), followed by two additional FC layers. The resulting protein embeddings from both pathways are subsequently refined through a two-layer BiLSTM network, with each layer containing 256 neurons to effectively capture bidirectional long-range dependencies. An overview of the model parameter settings is provided in Table S3.

The model development process involved a systematic optimization of various hyperparameters. Ultimately, a batch size of 32 and a learning rate of 0.0002 were selected. The Rectified Linear Unit (ReLU) was used as the activation function for the CNN and FC layers, while the hyperbolic tangent (Tanh) function was employed for the output activation in the BiLSTM components. To mitigate potential class imbalance, focal loss was incorporated into the training process. Throughout the training, model performance was closely monitored, with convergence assessed at the end of each epoch using a 5-fold cross-validation (CV) dataset. An early stopping mechanism was applied to identify the optimal model, ensuring an appropriate balance between underfitting and overfitting. The computational resources and training time details for LocPro are provided in Section Method S3 of Supplementary data.

### 2.3 Performance evaluation

To assess the model's overall performance, the F1 score and the area under precision-recall curve (AUPRC) as evaluation metrics. The F1 score combines precision and recall into a single measure, offering an effective evaluation of the model's performance, particularly in situations where balancing these metrics is crucial, such as in cases of class imbalance. The AUPRC quantifies the area under the precision-recall curve, emphasizing the trade-off between precision and recall in binary classification models. It is especially sensitive to the model's performance on positive class samples. To evaluate the model's performance for each subcellular localization, the Matthew's correlation coefficient (MCC) as a primary assessment metric. The MCC incorporates four critical parameters: true positives (TP), true negatives (TN), false positives (FP), and false negatives (FN). This comprehensive metric provides a balanced evaluation of the classifier's performance, particularly in the context of imbalanced datasets, thereby yielding more objective results. The relevant formulas and additional details are provided in Section Method S4 of Supplementary data.

### 2.4 Datasets construction

**Fig. 2:**
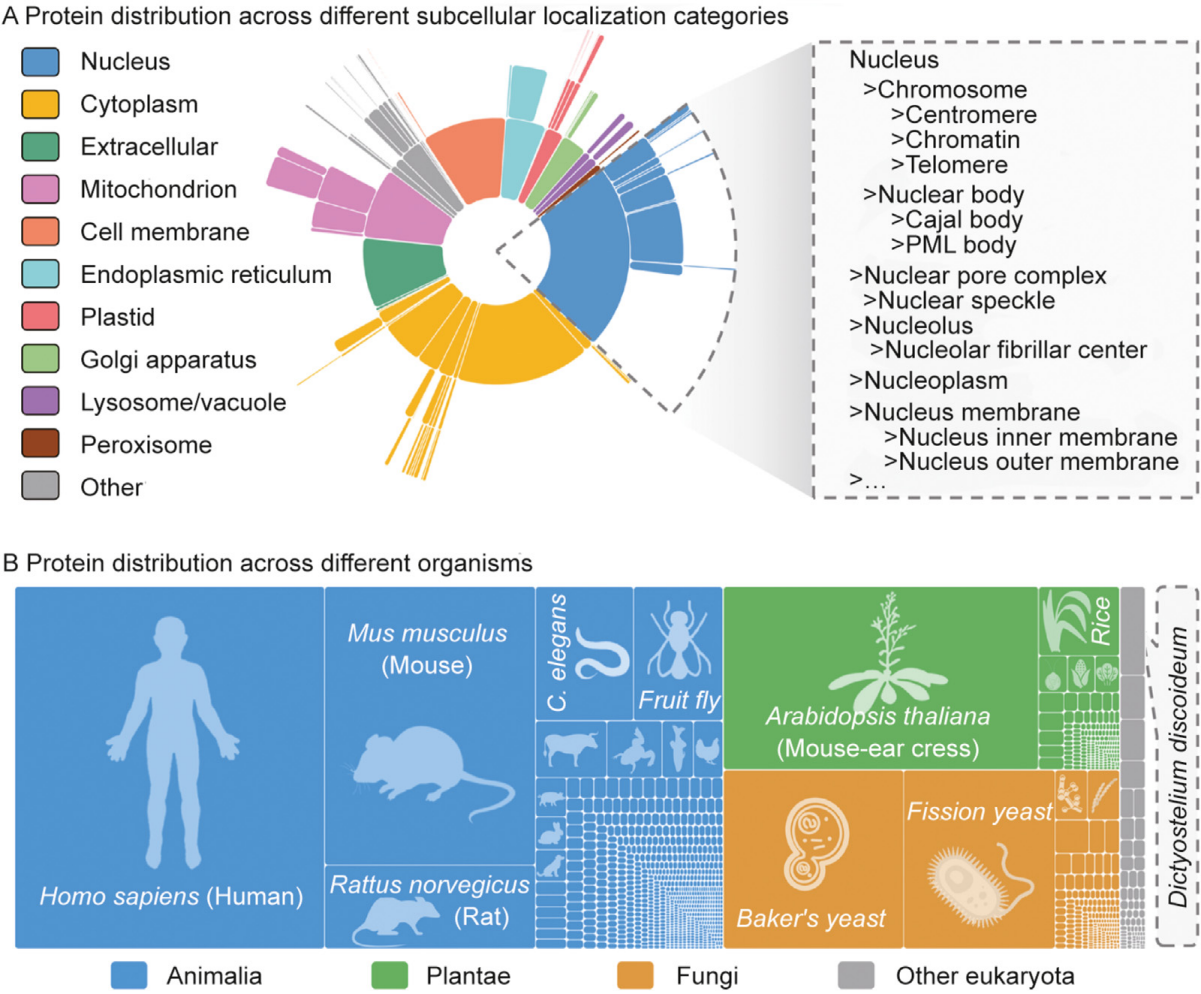
The distribution of subcellular localizations and organisms in the dataset constructed for this study. (A) Protein distribution across different subcellular localization categories. The dataset incorporates an extensive hierarchy of subcellular localization categories. For instance, the nuclear localization is further delineated into second-tier categories such as chromosome, nuclear body, and nucleus membrane. This categorization extends to a third tier, exemplified by localizations like centromere. (B) Protein distribution across different organisms. The dataset encompasses a wide range of species, representing diverse taxonomic groups. The Animalia kingdom includes organisms like *Homo sapiens* and *Mus musculus*. The Plantae kingdom includes organisms like *Arabidopsis thaliana* and *Oryza sativa* subsp. *Japonica*. The Fungi kingdom includes organisms like *Baker's yeast* and *Fission yeast*. The dataset also incorporates other eukaryotic organisms, exemplified by *Dictyostelium discoideum* and *Plasmopara viticola*. PML: promyelocytic leukemia.

The datasets utilized for LocPro were derived from the SubCELL database [33], which integrates high-confidence experimental annotations of protein subcellular localization from multiple sources, including UniProt, HPA, and others. The dataset construction process commenced with the extraction of raw protein subcellular localization data from SubCELL. Subsequently, protein sequence information was obtained by cross-referencing with UniProt. A total of 38,126 proteins were selected by choosing eukaryotic proteins and excluding any sequences with fewer than 40 amino acids. The distribution of these curated proteins across subcellular localizations and organisms is visually represented in Fig. 2. A distinctive characteristic of the dataset is its hierarchical structure of subcellular localization categories, providing a multi-level classification from broad to more specific localizations.

For this study, two multi-label prediction tasks were defined: Location-Main and Location-All. The Location-Main task aimed to predict 10 primary subcellular localizations, while the Location-All task predicted subcellular localization at multiple levels, resulting in 91 distinct localization categories, after retaining only those with more than 50 proteins. The dataset partitioning process was based on timestamps, with approximately 15% of the proteins allocated to an independent test set and the remaining 85% used for CV. The timestamp data were sourced from the “Date of last sequence modification” field in UniProt. For the CV dataset, a homology-based grouping method was employed to divide the proteins into five subsets for 5-fold CV. This was achieved using PSI-CD-HIT [34] with a 30% sequence identity threshold, clustering proteins based on sequence similarity. The resulting clusters were then evenly distributed across the five folds to ensure each fold represented a diverse subset of the data. This strategy was designed to minimize data leakage and reduce the risk of overfitting during model training, thereby improving the model's ability to generalize.

## 3 Results and discussion

### 3.1 Overview of a hybrid framework for protein subcellular localization prediction

In this study, a hybrid deep learning framework for predicting protein subcellular localization. As illustrated in Fig. 1A, the framework implements a sequential pipeline consisting of sequence input, feature representation, embedding integration, BiLSTM blocks, and localization prediction. The protein representation block, illustrated in Fig. 1B, integrates two complementary approaches: ESM2-derived representations, which capture complex sequence patterns through training on extensive protein data with strong generalization capabilities, and PROFEAT-engineered representations, which provide 1,484-dimensional expert knowledge features across seven distinct categories (detailed in Table S1). The PROFEAT-engineered protein representations undergo systematic processing (shown in Fig. 1C), including “Feature distance”, “Dimension reduction”, and “Coordinate assignment”, to map the protein representation onto a 2D grid. This approach effectively captures the intrinsic correlations among protein features, rather than treating them as entirely independent, facilitating a more effective integration of PROFEAT-based protein representations [35]. The consolidated features from both sources are processed through BiLSTM modules to extract bidirectional long-range dependencies, ultimately generating predictions for protein subcellular localization. The specifics of this novel deep learning framework are detailed in Section Materials and methods.

### 3.2 Comparing the overall performance among LocPro and existing tools

In this study, two distinct protein subcellular localization prediction tasks were established. The first task, Location-Main, focuses on predicting protein distribution across 10 major subcellular localizations. The second task, Location-All, expands the prediction scope to encompass all hierarchical levels of subcellular localization, accounting for varying degrees of spatial resolution within eukaryotic cells. To ensure robust model development and evaluation, two types of datasets were systematically organized: CV datasets (Location-Main-CV and Location-All-CV), which implement a 5-fold validation strategy, and independent test sets (Location-Main-Test and Location-All-Test) for unbiased performance assessment. The detailed procedures for dataset curation and organization are thoroughly documented in Section Materials and methods.

**Fig. 3:**
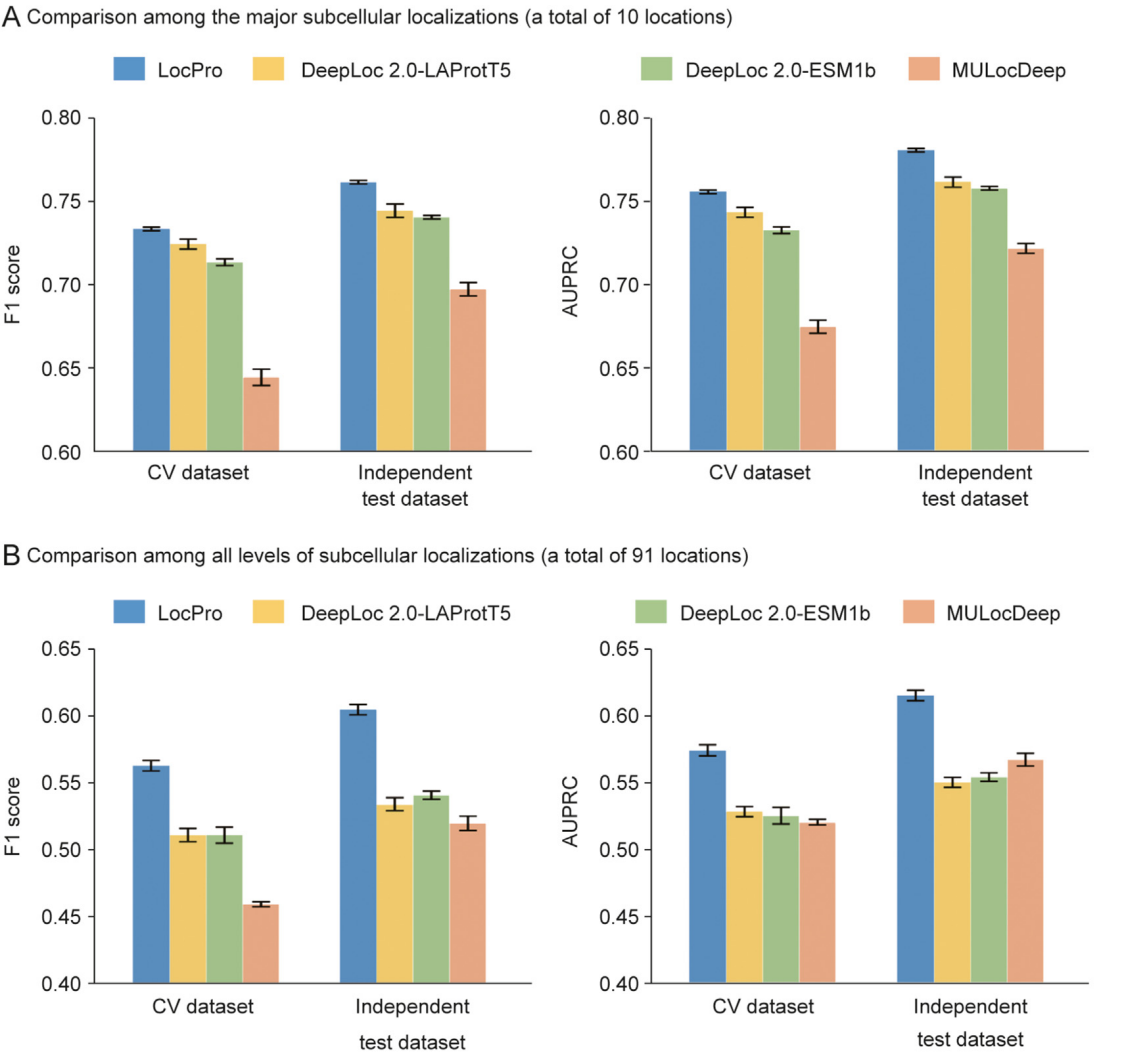
Comparison among the overall performances of LocPro and existing tools. (A) Comparison among the major subcellular localizations. The left and right panels present performance comparisons based on F1 score and the area under precision-recall curve (AUPRC), respectively. LocPro consistently outperforms other models across different test datasets and evaluation metrics while exhibiting minimal error bars, indicating better stability in prediction performance. (B) Comparison among all hierarchical levels of subcellular localization. LocPro demonstrates enhanced predictive capabilities compared to existing models, achieving a wider performance margin with improvements of 10.1% (F1 score) and 8.7% (AUPRC) in cross-validation (CV), and 11.8% (F1 score) and 8.4% (AUPRC) in independent testing. The specific values corresponding to this figure are detailed in Table S4.

The performance assessment of our proposed strategy involved systematic comparisons with established sequence-based prediction models. Specifically, LocPro was evaluated against two widely used tools for multi-label prediction of protein subcellular localization: DeepLoc 2.0 and MULocDeep. All models were trained using the datasets compiled in this study. The details of model replication are documented in Section Method S5 of Supplementary data. Performance evaluation was conducted using both CV sets and temporally segregated independent test sets. The results, including error bars indicating statistical variability, are visualized in Fig. 3, with detailed numerical values provided in Table S4. For the prediction of major subcellular localizations (Location-Main task), LocPro demonstrated superior performance across all evaluation metrics, including F1 score and AUPRC, on both CV and independent test sets, as illustrated in Fig. 3A. Moreover, in the more challenging task of predicting all hierarchical levels of subcellular localization (Location-All task), as illustrated in Fig. 3B, LocPro consistently outperformed existing methods across all metrics and test sets. Notably, compared to the second-best performing model, LocPro achieved significant improvements in the CV set (10.1% increase in F1 score and 8.7% increase in AUPRC) and the independent test set (11.8% increase in F1 score and 8.4% increase in AUPRC). Notably, our model demonstrated superior performance on independent test datasets compared to CV results, indicating that our protein homology-based dataset partitioning strategy and early stopping method (detailed in Section Materials and methods) effectively prevented data leakage and overfitting while enhancing generalization capability.

The study also evaluated and compared protein localization prediction across different eukaryotic kingdoms using independent test sets, with results detailed in Table S5. For the Location-Main task, performance improvements ranged from 2.2% to 5.2% in F1 score and from 2.7% to 5.1% in AUPRC. More substantial enhancements were observed in the Location-All task, with increases ranging from 3.8% to 14.2% in F1 score and from 3.5% to 7.4% in AUPRC. These significant performance gains across multiple evaluation metrics and datasets demonstrate the effectiveness of our novel LocPro strategy in advancing the field of eukaryotic protein subcellular localization prediction, while the consistent superior performance in both major localization and all-level localization tasks demonstrates its robust and generalizable prediction capabilities.

### 3.3 Comparing the localization-specific performance among LocPro and existing tools

Analyzing the localization-specific performance helps prevent poor performance in specific subcellular localizations from being masked by overall accuracy. Additionally, it provides readers with valuable insights into the confidence levels of predictions across different subcellular localizations. In this study, the performance of various models in predicting protein localization at each subcellular location was further analyzed. Therefore, based on the Location-Main task, the prediction capabilities of different models across 10 major subcellular locations were compared, as summarized in Table 1. Our results show that LocPro achieved the best performance in seven out of the ten subcellular locations, including the nucleus, cytoplasm, extracellular, cell membrane, endoplasmic reticulum, golgi apparatus, and lysosome/vacuole. Additionally, LocPro ranked second in performance for the remaining three locations.

**Table 1:** Localization-specific performances across major locations.

Locations	LocPro	DeepLoc 2.0 -LAProtT5	DeepLoc 2.0 -ESM1b	MULocDeep
Cell membrane	**0.662±0.006**	0.649 ± 0.003	0.645 ± 0.005	0.596 ± 0.007
Cytoplasm	**0.612±0.002**	0.584 ± 0.002	0.601 ± 0.003	0.488 ± 0.014
Endoplasmic reticulum	**0.533±0.007**	0.475 ± 0.007	0.469 ± 0.021	0.454 ± 0.007
Extracellular	**0.892±0.003**	0.883 ± 0.003	0.875 ± 0.006	0.865 ± 0.004
Golgi apparatus	**0.373±0.003**	0.337 ± 0.012	0.303 ± 0.006	0.226 ± 0.010
Lysosome/vacuole	**0.341±0.027**	0.267 ± 0.007	0.277 ± 0.014	0.145 ± 0.014
Mitochondrion	0.754 ± 0.003	0.751 ± 0.004	**0.778±0.002**	0.690 ± 0.004
Nucleus	**0.718±0.002**	0.681 ± 0.003	0.712 ± 0.001	0.611 ± 0.004
Peroxisome	0.524 ± 0.017	**0.550±0.003**	0.450 ± 0.013	0.105 ± 0.054
Plastid	0.816 ± 0.003	0.814 ± 0.004	**0.845±0.005**	0.712 ± 0.006

This table uses Matthew's correlation coefficient (MCC) as the performance evaluation metric for per localization, and presents mean values ± standard error derived from independent testing. The values representing the highest performance metrics across all methods were denoted in bold, while the second-highest values are underlined.

Moreover, it was observed that certain subcellular localization tasks were relatively easier to predict than others. The Extracellular and Plastid localization tasks are relatively straightforward due to the distinct features of their associated proteins. Extracellular proteins often have signal peptides that direct their secretion outside the cell [36], and the clearly defined extracellular space minimizes overlap with intracellular compartments, simplifying classification. Similarly, plastid proteins contain transit peptides that guide their transport to plastids [37], such as chloroplasts. As well-characterized organelles with specific functions like photosynthesis, plastids house proteins with unique features, facilitating accurate prediction. In contrast, the tasks involving the Golgi apparatus and Lysosome/vacuole proved more challenging, likely due to the complexity, multifunctionality, dynamic nature, and frequent interactions of these organelles with other cellular components [38,39]. In addition, by analyzing the relationship between the MCC values and the sample size rankings of subcellular localization categories (shown in Fig. S1), it is evident that there is no direct correlation between prediction performance and sample size. This further indicates that the model's predictive performance across these categories is not simply driven by sample size.

In the Location-All task, model performance was evaluated across mitochondrial and sub-mitochondrial compartments. Mitochondria, often referred to as the cellular powerhouse, are responsible for producing adenosine triphosphate (ATP), regulating cellular metabolism, and participating in processes such as apoptosis [[40], [41], [42]]. Therefore, studying the protein localization within mitochondria is highly valuable. As shown in Table 2, LocPro achieved the best performance in predicting the mitochondrion, mitochondrion matrix, mitochondrion membrane, and mitochondrion inner membrane, while it attained second-best performance in predicting the mitochondrion outer membrane. These analyses demonstrate that LocPro consistently achieves optimal or near-optimal performance across various subcellular localizations, further validating the effectiveness and stability of our proposed strategy for protein subcellular localization prediction.

**Table 2:** Performance across different mitochondrial localizations.

Locations	LocPro	DeepLoc 2.0 -LAProtT5	DeepLoc 2.0 -ESM1b	MULocDeep
Mitochondrion	**0.762±0.003**	0.753 ± 0.005	0.733 ± 0.004	0.690 ± 0.006
Mitochondrion matrix	**0.701±0.010**	0.657 ± 0.002	0.589 ± 0.009	0.544 ± 0.011
Mitochondrion membrane	**0.678±0.004**	0.579 ± 0.014	0.554 ± 0.010	0.502 ± 0.023
Mitochondrion inner membrane	**0.693±0.012**	0.602 ± 0.008	0.539 ± 0.019	0.466 ± 0.025
Mitochondrion outer membrane	0.450 ± 0.032	0.286 ± 0.033	**0.451±0.016**	0.254 ± 0.096

This table uses Matthew's correlation coefficient (MCC) as the performance evaluation metric for per localization, and presents mean values ± standard error derived from independent testing. The values representing the highest performance metrics across all methods were denoted in bold, while the second-highest values are underlined.

### 3.4 Subcellular localization prediction of drug targets

Understanding the subcellular localization of drug targets is crucial for drug development. Knowledge of target subcellular localization can be applied to target assessment and recommendations [43], guiding the optimization of drug properties [44], understanding the toxic mechanisms of drugs [45], and directing the development of organelle-targeted drug delivery systems [46]. To evaluate the predictive capability of LocPro, drug targets were collected from the Therapeutic Target Database [47,48] and curated their subcellular localization information using data from additional database. A total of 1,994 validated proteins distributed across 9 major subcellular localizations, were compiled, and the predictions from LocPro were systematically compared with their experimentally verified subcellular localizations. For proteins that did not meet the threshold for any specific subcellular localization label, the model assigned them to the label closest to the threshold.

As shown in Table 3, in predicting the subcellular localization of drug targets, LocPro achieved an overall recall of 67.0% and an overall precision of 88.4%. The highest recall was observed for Cell membrane proteins. Notably, the precision exceeded 90% for proteins localized in the mitochondrion, nucleus, and Cell membrane. For the three locations (cytoplasm, cell membrane, and nucleus) containing the largest number of drug targets, the model demonstrated robust performance with recall rates of 69.62%, 81.36%, and 65.72%, and precision rates of 86.04%, 90.65%, and 91.70%, respectively. These results demonstrate that our proposed model can efficiently predict the subcellular localization of drug targets, providing valuable guidance for drug research and development.

**Table 3:** Subcellular localization prediction of drug targets.

Locations	True count	Predicted count	Hit count	Recall (hit/true, %)	Precision (hit/predicted, %)
Cell membrane	751	674	611	81.36	90.65
Cytoplasm	859	695	598	69.62	86.04
Endoplasmic reticulum	163	90	79	48.47	87.78
Extracellular	236	194	165	69.92	85.05
Golgi apparatus	105	14	11	10.48	78.57
Lysosome/Vacuole	63	45	26	41.27	57.78
Mitochondrion	190	111	107	56.32	96.40
Nucleus	706	506	464	65.72	91.70
Peroxisome	11	6	4	36.36	66.67
**Total**	**3084**	**2335**	**2065**	**66.96**	**88.44**

True count represents the actual number of targets in each location. Predicted count indicates the number of targets predicted in each location by LocPro. Hit count denotes the number of targets that are correctly predicted and match the actual targets in each location.

### 3.5 Availability of LocPro web server

In this study, a web server, LocPro, was developed to provide easy access to the subcellular localization prediction method. The server is hosted on a CentOS Linux platform, using Nginx as the web server. The backend is built with Python and the Django framework, while the frontend utilizes Vue.js with Element UI components. The visualization of subcellular localizations is achieved using SwissBioPics [49]. This open-access platform is compatible with major web browsers, including Chrome, Firefox, Edge, and Safari, and is freely accessible at https://idrblab.org/LocPro/.

**Fig. 4:**
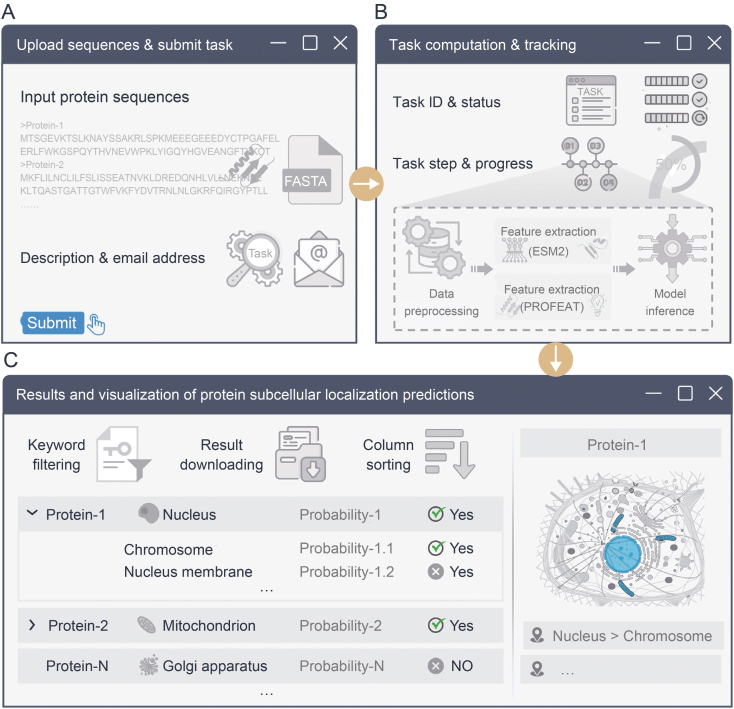
Summary flowchart of the LocPro webserver. (A) Upload sequences and submit task. Users are required to upload protein sequences in FASTA format. Additionally, users can optionally describe the prediction task and provide an email address to receive the results. (B) Task computation and tracking. Upon task submission, the task is placed in a queue for processing on the server backend. Users can monitor the real-time progress of their tasks using the task ID. Each task follows a four-step process: data preprocessing, feature extraction (ESM2), feature extraction (PROFEAT), and model inference. (C) Results and visualization of protein subcellular localization predictions. The results for each task are displayed in a table, featuring interactive options such as keyword filtering, result downloading, and column sorting to enhance user experience. Users can click the leftmost button to view predictions for more specific subcellular locations. Additionally, the web server offers visualization tools to depict the predicted subcellular localization of selected protein.

The LocPro webserver workflow comprises three main stages as shown in Fig. 4. In the first stage, users submit protein sequences in FASTA format along with optional parameters such as task description and email address for result notifications. During the second stage, the submitted task enters the server's computational queue where users can track task progress in real time using the assigned task ID. In the final stage, prediction results are presented in a comprehensive table with interactive features including Keyword filtering, Result downloading, and Column sorting. Users can access detailed predictions for specific subcellular locations via the leftmost button, and interactive visualization tools are available to display predicted protein localizations.

## 4 Conclusion and perspectives

In this study, LocPro, a deep learning framework for predicting protein subcellular localization, was presented. The major contributions of this work are as follows: (a) the integration of protein representations derived from the pre-trained large language model (LLM) ESM2 and the expert-driven tool PROFEAT, (b) the implementation of a hybrid deep neural network architecture that combines CNN, FC, and BiLSTM blocks, and (c) the development of a multi-label framework for predicting protein subcellular localization at multiple granularity levels. Comparative experiments with popular models demonstrated the strong performance of LocPro, establishing it as an effective tool for protein subcellular localization prediction.

However, it is recognized that protein subcellular localization is influenced by a variety of factors beyond the primary sequence, including post-translational modifications, protein-protein interactions, and the cellular environment and state. Therefore, future research could focus on integrating different types of data to further enhance the accuracy and robustness of localization predictions. Additionally, understanding the mislocalization of proteins caused by mutations, as well as the localization of viral proteins within host cells, offers important insights for advancing research into disease mechanisms and therapeutic development. Therefore, subsequent models could focus on improving prediction performance in these areas to provide deeper insights into the complex biological processes governing protein subcellular localization.

## CRediT authorship contribution statement

**Yintao Zhang:** Writing – original draft, Visualization, Methodology, Data curation, Software, Writing – review & editing. **Lingyan Zheng:** Methodology, Writing – original draft. **Nanxin You:** Methodology, Investigation, Visualization. **Wei Hu:** Data curation, Methodology, Visualization. **Wanghao Jiang:** Data curation, Visualization. **Mingkun Lu:** Methodology. **Hangwei Xu:** Data curation. **Haibin Dai:** Writing – review & editing, Supervision. **Tingting Fu:** Funding acquisition, Supervision, Writing – review & editing. **Ying Zhou:** Writing – review & editing, Methodology, Conceptualization, Data curation, Writing – original draft.

## Declaration of competing interest

The authors declare that there are no conflicts of interest.
