# Supplementary material for: LocPro: A deep learning-based prediction of protein subcellular localization for promoting multi-directional pharmaceutical research
Source: J Pharm Anal. 2025 Mar 5;15(8):101255. doi: 10.1016/j.jpha.2025.101255 (PMC12363569; doi:10.1016/j.jpha.2025.101255)
Supplement: Multimedia component 1 [file mmc1.pdf]

LocPro: a deep learning-based prediction of protein subcellular localization for promoting multi-directional pharmaceutical research

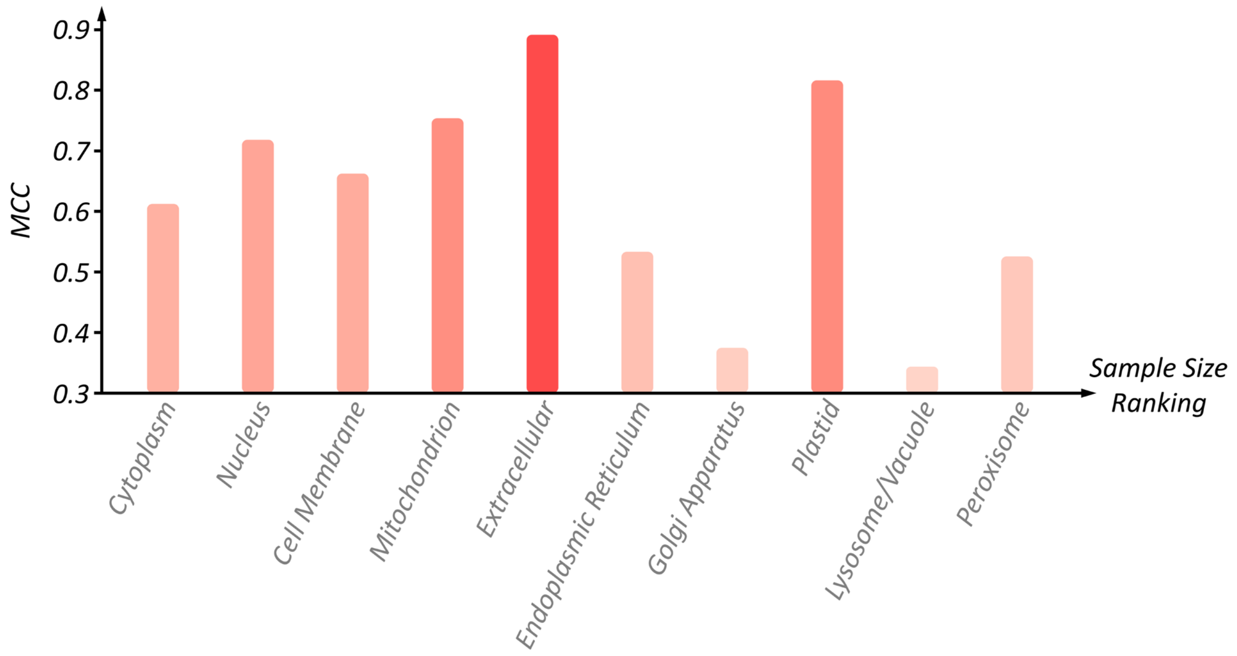

Fig. S1. Relationship between model performance and sample size rankings of subcellular localization categories. The y-axis represents the MCC values, while the x-axis shows the rankings for various subcellular localization categories. Categories with smaller sample sizes are positioned further to the right on the x-axis. The color intensity of the bars indicates the magnitude of MCC values, with darker bars representing higher MCC values. The figure illustrates that there is no direct correlation between model performance and sample size.

Table S1. PROFEAT-based feature engineering for generating 1484-dimensional features categorized into seven groups

Feature Categories	Number of Features	Feature Description
2D-Scatter APAAC	80	Amphiphilic Pseudo amino acid composition
Autocorrelation	270	Autocorrelation descriptors
Composition	20	Amino Acid Composition
	400	Dipeptide Composition
Interaction	21	CTD according to Amino acid flexibility index
	21	CTD according to charge
	21	CTD according to CLogP
	21	CTD according to hydrophobicity
	21	CTD according to Molecular weight
	21	CTD according to No. of hydrogen bond acceptor in side chain
	21	CTD according to No. of hydrogen bond donor in side chain
	21	CTD according to normalized vdW volumes
	21	CTD according to polarity
	21	CTD according to polarizability
	21	CTD according to secondary structure
	21	CTD according to solubility in water
	21	CTD according to solvent accessibility
	21	CTD according to Surface tension
PAAC for AA Index	50	PAAC for amino acid index set

Physiochemical	21	CTD according to propensity for Protein-ligand polar and arom-Imai
	21	CTD according to Protein-DNA Interface propensity-Ahmad
	21	CTD according to Protein-DNA Interface propensity-Schneider
	21	CTD according to Protein-ligand binding site propensity-Khazanov
	21	CTD according to Protein-ligand valid binding site propen-Khazanov
	21	CTD according to Protein-protein Interface (PPI) propensity-Ma
	21	CTD according to Protein-protein Interface hotspot propensity-Bogan
	21	CTD according to Protein-RNA Interface propensity-Ellis
	21	CTD according to Protein-RNA Interface propensity-Kim
	21	CTD according to Protein-RNA Interface propensity-Phipps
QSO Descriptors	160	Quasi-sequence-order descriptors

Table S2. Impact of segment length on model performance

Datasets	Assessments	Length 500	Length1000	Length 2000
Location-Main (*CV*)	F1 Score	0.727±0.002	0.731±0.001	0.734±0.001
	AUPRC	0.749±0.001	0.752±0.001	0.756±0.001
Location-Main (*test*)	F1 Score	0.758±0.002	0.760±0.003	0.762±0.001
	AUPRC	0.777±0.002	0.778±0.003	0.781±0.001
Location-All (*CV*)	F1 Score	0.550±0.005	0.558±0.005	0.565±0.004
	AUPRC	0.562±0.005	0.569±0.005	0.576±0.004
Location-All (*test*)	F1 Score	0.585±0.004	0.588±0.004	0.607±0.004
	AUPRC	0.595±0.004	0.598±0.003	0.617±0.004

The table presents mean values ± standard error derived from 5-fold cross-validation, as well as independent testing.

Table S3. Model hyperparameter configurations

Hyperparameter Type	Description	Setting
Number of CNN Layers	Number of convolutional layers	2
Kernel Size (CNN)	Kernel size for the first convolutional layer	3×3
Padding (CNN)	Padding for the first convolutional layer	1
Max Pooling Kernel Size (Pool)	Kernel size for the first max-pooling layer	2×2
Max Pooling Stride (Pool)	Stride for the first max-pooling layer	2
Number of FC Layers	Number of fully connected layers	3
Input Size (Dense3)	Input size to the concatenation layer	2048
Output Size (Dense3)	Output size of the concatenation layer	512
Number of LSTM Layers	Number of LSTM layers	2
Hidden Size (LSTM)	Number of hidden units in each LSTM layer	256
Bidirectional (LSTM)	Whether the LSTM is bidirectional	TRUE
Dropout Rate	Dropout rate applied after fully connected layers	0.5
Activation (CNN and FC)	Activation function used in CNN and FC layers	ReLU
Activation (LSTM)	Activation function used in LSTM output	Tanh
Sequence Length (LSTM)	Length of the sequence input to LSTM	11
Batch Size	Size of the batch during training	32
Learning Rate	Learning rate for the optimizer	0.0002
Loss Function	Loss function used during training	Focal Loss
Early Stopping	Epochs for Early Stopping	5

Table S4. Overall performance among LocPro and existing tools

Datasets	Assessments	LocPro	DeepLoc 2.0 -LAProtT5	DeepLoc 2.0 -ESM1b	MULocDeep
Location-Main (*CV*)	F1 Score	**0.734±0.001**	0.725±0.003	0.714±0.002	0.645±0.005
	AUPRC	**0.756±0.001**	0.744±0.003	0.733±0.002	0.675±0.004
Location-Main (*test*)	F1 Score	**0.762±0.001**	0.745±0.004	0.741±0.001	0.698±0.004
	AUPRC	**0.781±0.001**	0.762±0.003	0.758±0.001	0.722±0.003
Location-All (*CV*)	F1 Score	**0.565±0.004**	0.513±0.005	0.513±0.006	0.461±0.002
	AUPRC	**0.576±0.004**	0.530±0.004	0.527±0.006	0.522±0.002
Location-All (*test*)	F1 Score	**0.607±0.004**	0.536±0.005	0.543±0.003	0.522±0.005
	AUPRC	**0.617±0.004**	0.552±0.004	0.556±0.003	0.569±0.005

The table presents mean values ± standard error derived from 5-fold cross-validation, as well as independent testing. The values representing the highest performance metrics across all methods were denoted in **bold**, while the second-highest values are underlined.

Table S5. Overall Performance across different biological kingdoms

Datasets	Assessments	LocPro	DeepLoc 2.0 -LAProtT5	DeepLoc 2.0 -ESM1b	MULocDeep
Location-Main (*Animalia test*)	F1 Score	**0.779±0.001**	<u>0.762±0.003</u>	0.760±0.001	0.724±0.005
	AUPRC	**0.803±0.001**	<u>0.782±0.003</u>	0.780±0.001	0.752±0.004
Location-Main (*Plantae test*)	F1 Score	**0.742±0.002**	<u>0.713±0.005</u>	0.706±0.003	0.653±0.007
	AUPRC	**0.768±0.001**	<u>0.733±0.005</u>	0.726±0.003	0.686±0.006
Location-Main (*Fungi test*)	F1 Score	**0.789±0.002**	<u>0.750±0.005</u>	0.739±0.002	0.703±0.006
	AUPRC	**0.812±0.002**	<u>0.773±0.004</u>	0.762±0.002	0.734±0.004
Location-All (*Animalia test*)	F1 Score	**0.610±0.006**	0.528±0.006	<u>0.534±0.003</u>	0.523±0.005
	AUPRC	**0.622±0.005**	0.549±0.005	0.552±0.003	<u>0.579±0.005</u>
Location-All (*Plantae test*)	F1 Score	**0.661±0.005**	0.629±0.005	<u>0.637±0.007</u>	0.590±0.003
	AUPRC	**0.684±0.004**	0.653±0.005	<u>0.661±0.007</u>	0.630±0.003
Location-All (*Fungi test*)	F1 Score	**0.763±0.002**	<u>0.712±0.004</u>	0.696±0.004	0.627±0.014
	AUPRC	**0.784±0.002**	<u>0.735±0.003</u>	0.721±0.004	0.667±0.012

The table presents mean values ± standard error derived from 5-fold cross-validation, as well as independent testing. The values representing the highest performance metrics across all methods were denoted in **bold**, while the second-highest values are <u>underlined</u>.

Method S1. Global representation method for amino acid sequences based on ESM2

The ESM2 (Evolutionary Scale Modeling 2) method leverages a protein large language model (LLM) to process protein sequences and generate per-residue embeddings. A key advantage of the ESM2 model is its ability to learn from large-scale protein data, enabling it to generalize well across a wide range of protein sequences. This makes it particularly useful for tasks like protein classification, structure prediction, and functional annotation, as it integrates evolutionary information to improve predictive accuracy. By training on a diverse set of protein sequences, ESM2 learns context-dependent relationships between amino acids, which is critical for capturing the complex dependencies present in protein sequences.

This method takes an amino acid sequence $X = \{x_1, x_2, x_3, \dots, x_n\}$ and outputs a set of embeddings $E \in \mathbb{R}^{d \times n} = \{e_1, e_2, e_3, \dots, e_n\}$, where each embedding $e_i \in \mathbb{R}^d$ represents the ith residue. These embeddings capture both the local and global sequence information, allowing for a more nuanced representation of protein structures compared to traditional methods. To standardize the size of the representations, particularly for sequences of varying lengths, a pooling step is applied. This is typically done by averaging the embeddings along the sequence length. The global representation E_{global} is computed as the mean of the per-residue embeddings:

$$E_{global} = \frac{1}{n} \times \sum_{i=1}^{n} e_i$$

This average pooling approach ensures that the entire sequence is represented by a fixed-size vector, regardless of its length, allowing for downstream analysis and applications. The integration of sequence-wide information through pooling further enhances the ability of ESM2 to capture global sequence patterns, making it a powerful tool for understanding protein function and structure.

or particularly long protein sequences, a segmentation approach was employed. Specifically, the entire protein sequence X is divided into N non-overlapping segments, denoted as $X_1, X_2, \dots, X_N$. For each segment X_i, with length L_i, an embedding E_i of 1280 dimensions is generated using the ESM2 model. The final embedding E for the entire sequence is obtained by taking a weighted average of these segment embeddings, as given by the formula:

$$E = \frac{\sum_{i=1}^{N} L_i E_i}{\sum_{i=1}^{N} L_i}$$

Additionally, the impact of different segment lengths on model performance was evaluated (as shown in **Table S2**). The results indicate that as the segment length increases, there is a slight improvement in model performance, although the magnitude of this improvement is not substantial. In this study, a segment length of 2000 was used for the model. However, the LocPro web server employs a segment length of 500 to balance computational efficiency and performance.

Method S2. Methods for constructing PROFEAT similarity-based images (PF-IMG)

Min-Max Normalization is a data normalization technique that scales the values of a dataset to a fixed range, typically [0, 1]. It transforms each feature by subtracting the minimum value of the feature and dividing by the range. This process ensures that all values are within the desired range, which is particularly useful for algorithms that depend on the scale of input features, such as machine learning models. For a given feature X_{ij} in this matrix, where X_i denotes the ith feature, X_i^{min} and X_i^{max} represent the minimum and maximum values of the ith across all proteins, the normalized value X_{ij}^{norm} is computed as:

$$X_{ij}^{norm} = \frac{X_{ij} - X_i^{min}}{X_i^{max} - X_i^{min}}$$

Cosine Distance is a metric used to assess the dissimilarity between two vectors by measuring the cosine of the angle between them. *Cosine distance* is particularly valuable for evaluating the similarity or dissimilarity between feature vectors. In this study, the feature distance matrix was constructed by calculating pairwise cosine distances among the 1,484 features. For two feature vectors X_i and X_j, the *Cosine distance* is computed using the following formula:

$$Distance(X_i, X_j) = 1 - \frac{X_i \cdot X_j}{\|X_i\| \, \|X_j\|}$$

UMAP works by constructing a weighted graph of the data in the high-dimensional space based on local neighborhood relationships, and then tries to find a low-dimensional representation of the data that preserves these relationships. It operates in two main stages: first, it constructs a topological representation of the data in the original space using fuzzy simplicial sets, and second, it optimizes the low-dimensional representation by minimizing the difference between the original and projected graphs.

The *J-V algorithm* is used to optimize the placement of points in a 2D space, commonly for multidimensional scaling, clustering, or visualizing high-dimensional data. It efficiently assigns coordinates to data points in a 2D grid based on predefined distances, aiming to minimize the distortion between the high-dimensional and 2D distances. This is achieved by iteratively adjusting the point positions to reduce the discrepancy between the original and projected distances.

Method S3. Computational resources and training time

In this study, the ESM2 version *esm2_t33_650M_UR50D* was used for protein sequence characterization. Due to the significant increase in memory requirements as the protein length increases, a two-step approach was adopted to manage computational resources effectively. First, a CPU was used to generate the embeddings for all protein sequences. This step was necessary to handle the high memory demands of ESM2, which can be challenging for GPUs with limited video memory. Subsequently, an NVIDIA RTX 3080 GPU with 10 GB of video memory was utilized for the training of the LocPro model. The training time for the model was approximately 2 hours.

Method S4. Details of model performance evaluation methods

The F1 score integrates *precision* and *recall*, providing a single measure that effectively evaluates the model's overall performance, particularly in scenarios where a balance between these metrics is essential, such as in cases of class imbalance. The formula for the F1 score is as follows:

$$F1 = 2 \times \frac{Precision \times Recall}{Precision + Recall}$$

The AUPRC quantifies the *area under the precision-recall curve*, focusing on the trade-off between *precision* and *recall* for binary classification models, and is particularly sensitive to the model's predictive performance on positive class samples.

The formula for the *precision* is as follows:

$$Precision = \frac{TP}{TP + FP}$$

The formula for the *recall* is as follows:

$$Recall = \frac{TP}{TP + FN}$$

The *Matthew's correlation coefficient* (MCC) as a primary assessment metric. The MCC incorporates four critical parameters: *true positives* (TP), *true negatives* (TN), *false positives* (FP), and *false negatives* (FN). This comprehensive metric provides a balanced evaluation of the classifier's performance, particularly in the context of imbalanced datasets, thereby yielding more objective results. The formula for the MCC is as follows:

$$MCC = \frac{TP \times TN - FP \times FN}{\sqrt{(TP + FP)(TP + FN)(TN + FP)(TN + FN)}}$$

Method S5. The processes of existing methods for model construction

In this study, a comprehensive comparative analysis of the proposed model against leading multi-label prediction tools for eukaryotic protein subcellular localization, namely DeepLoc 2.0 and MULocDeep. To ensure an equitable evaluation, rigorous training and testing protocols, as outlined in the **Materials and Methods** section. The comparative analysis involved retraining all models using two custom-constructed datasets: *Location-Main* and *Location-All*. DeepLoc 2.0 employs two distinct protein representation strategies, represented by its variants: DeepLoc 2.0-LAProtT5 and DeepLoc 2.0-ESM1b. Additionally, for MULocDeep, its original alternating training mode was specifically designed for its curated dataset and could not be directly applied to the datasets provided in this study. To ensure methodological consistency and a fair comparison across all models, an independent training strategy for each model using the aforementioned datasets was implemented. A consistent evaluation framework was also established and maintained to facilitate an objective performance assessment. This standardized approach enabled fair and systematic comparisons between different model architectures and methodologies. By incorporating these state-of-the-art deep learning-based approaches and conducting experiments in a standardized manner, our study aims to provide a comprehensive and robust evaluation of various methodologies for protein subcellular localization prediction.
